# Health-Related Quality of Life Trajectories across 10 Years after Moderate to Severe Traumatic Brain Injury in Norway

**DOI:** 10.3390/jcm10010157

**Published:** 2021-01-05

**Authors:** Marit V. Forslund, Paul B. Perrin, Solrun Sigurdardottir, Emilie I. Howe, Marleen R. van Walsem, Juan Carlos Arango-Lasprilla, Juan Lu, Alba Aza, Tone Jerstad, Cecilie Røe, Nada Andelic

**Affiliations:** 1 Department of Physical Medicine and Rehabilitation, Oslo University Hospital, 0424 Oslo, Norway; e.i.howe@medisin.uio.no (E.I.H.); cecilie.roe@medisin.uio.no (C.R.); nandelic@online.no (N.A.); 2 Departments of Psychology and Physical Medicine and Rehabilitation, Virginia Commonwealth University, Richmond, VA 23233, USA; pperrin@vcu.edu; 3 Centre for Rare Disorders, Oslo University Hospital, 0424 Oslo, Norway; sosigu@ous-hf.no; 4 Institute of Clinical Medicine, Faculty of Medicine, University of Oslo, 0318 Oslo, Norway; 5 Department of Neurohabilitation, Oslo University Hospital, 0424 Oslo, Norway; r.m.van.walsem@medisin.uio.no; 6 Department of Neurology, Oslo University Hospital, 0424 Oslo, Norway; 7 Research Centre for Habilitation and Rehabilitation Models and Services (CHARM), Institute of Health and Society, Faculty of Medicine, University of Oslo, 0318 Oslo, Norway; 8 Biocruces Bizkaia Health Research Institute, 48903 Barakaldo, Spain; jcalasprilla@gmail.com; 9 IKERBASQUE, Basque Foundation for Science, 48013 Bilbao, Spain; 10 Department of Cell Biology and Histology, University of the Basque Country (UPV/EHU), 48940 Leioa, Spain; 11 Department of Family Medicine and Population Health, Division of Epidemiology, Virginia Commonwealth University, Richmond, VA 23233, USA; juan.lu@vcuhealth.org; 12 Faculty of Psychology, University of Salamanca, 37005 Salamanca, Spain; azhernandez@usal.es; 13 Institute on Community Inclusion (INICO), University of Salamanca, 37005 Salamanca, Spain; 14 Department of Neuroradiology, Oslo University Hospital, 0424 Oslo, Norway; uxtoad@ous-hf.no

**Keywords:** traumatic brain injury, outcome assessment, physical health, mental health, SF-36, longitudinal studies, rehabilitation

## Abstract

Traumatic brain injury (TBI) has a long-lasting impact on participation and health-related quality of life (HRQL). We aimed to describe the physical and mental health trajectories and to identify their predictors across the first 10 years after TBI. A prospective longitudinal cohort of 97 individuals with moderate to severe TBI (age 16–55 years) in Norway were followed up at 1, 2, 5, and 10 years post-injury. Their socio-demographic and injury characteristics were recorded at baseline; their responses to the 36-Item Short Form Health Survey (SF-36) were collected at each follow-up. The Physical (PCS) and Mental Component Summary (MCS) scores were used as the outcome measures of physical and mental health. The predictors of the trajectories were described and examined using hierarchical linear modelling. The subscale scores showed a stable or increasing trend, but only the Role Physical and Role Emotional subscales showed clinically relevant positive changes from 1 to 10 years post-injury. Longer time, male gender, employment pre-injury, and shorter length of post-traumatic amnesia were significant predictors of better physical health trajectories; longer time, male gender, and employment pre-injury were significant predictors of better mental health trajectories. At-risk individuals may be targeted to receive rehabilitation interventions to improve their long-term quality of life outcomes.

## 1. Introduction

Moderate and severe traumatic brain injury (TBI) can lead to long-lasting functional impairment, profoundly influencing the lives of those who are affected. Physical, cognitive, and emotional changes, and inability to return to pre-injury activities affect participation and health-related quality of life (HRQL) [1,2]. HRQL is a multidimensional concept referring to “the direct impact of a medical condition or treatment on an individual’s perception of their physical, emotional or social well-being” [3].

Every year in Europe alone, approximately 2.5 million people sustain a TBI [2]. With the increasing survival rate after TBI, focus has shifted to treatment and rehabilitation in the post-acute and chronic phases [2]. Disabilities resulting from TBI are complex and may have long-lasting consequences for the patient’s functioning and well-being. Clinical follow-up and rehabilitation programs use standardized measures to track the patient’s functional level and provide adequate interventions and healthcare services. As improving well-being and HRQL are key goals of most health interventions and the ultimate goal of TBI rehabilitation, the importance of including both objective clinical indicators and HRQL measures when assessing outcomes has increasingly been acknowledged [4]. HRQL is assessed by generic or disease-specific measures [4,5,6]. Generic measures allow for comparison with the general population as well as other medical conditions, while disease-specific measures are more sensitive to the attributes of a specific condition. Within the field of TBI, the Medical Outcomes Scale 36-Item Short-Form Health Survey (SF-36) [7] is a widely used measure of HRQL. It has been established as a valid and reliable generic instrument for capturing the subjective perspective of individuals with TBI [4,8,9].

Studies using the SF-36 to assess HRQL in individuals with TBI have broadly found a decreased HRQL compared to the general population [3,10,11,12,13,14]. Several long-term follow-up studies have shown a lower HRQL compared to the general population a decade after injury [10,15], whereas others have shown improvement over time to reach normal population levels [16,17,18].

The factors most consistently associated with lower HRQL measured by the SF-36 in individuals with TBI are depressive symptoms [10,16,17,18,19,20,21,22], unemployment and lower productivity [10,15,16,21,23], a lower functional level (measured by the Glasgow Outcome Scale [GOS] or the extended GOS [GOSE]) [10,18,24], poorer community integration and social support [20,25], a higher number of post-concussive symptoms [12,26], and worse cognitive impairment [14]. Demographic factors, such as age, education level, marital status, and gender, have shown contradictory results. While some studies have found an association between female gender and lower HRQL [10,16,25,27,28], others have found that female gender is associated with higher HRQL [15,21]. Studies on measures of injury severity have yielded inconclusive results, with some studies reporting an association between more severe injury and lower HRQL in individuals with TBI [20,27,28], but other studies could not find this relationship [10,11,15,18].

Although providing valuable information on HRQL and the associated socio-demographic and injury characteristics, the current literature is limited to a majority of retrospective studies, mixed study populations, lack of long-term follow-ups, and small sample sizes. To date, there is a lack of research on the long-term trajectories of HRQL over 10 years after TBI. Identifying the socio-demographic and clinical characteristics that can predict the long-term trajectories of HRQL is important as it may help identify individuals who are at risk of poorer outcomes and in need of long-term rehabilitation efforts. To achieve this goal, hierarchical linear modelling (HLM) is an appropriate statistical strategy for outcomes with repeated measurements. It is stronger in terms of statistical power than traditional regression analyses, as it provides a better estimate of standard errors and is robust in managing missing data [29].

The present study is a 10-year extension of a longitudinal cohort, and previous publications on HRQL include the SF-36 outcome at 1 and 2 years after injury [20,30] and an examination of the physical functioning domains up to 5 years after injury [31]. The aims of the present study were to (1) describe the changes in health-related functioning and well-being along eight SF-36 subscales up to 10 years after injury; (2) assess the physical and mental health trajectories in individuals with moderate to severe TBI at 1, 2, 5, and 10 years post-injury; and (3) investigate whether socio-demographics and injury severity characteristics can predict these trajectories.

Based on previous research, we hypothesized that the SF-36 subscale scores and component summary scale scores would increase over time and that employment pre-injury, gender, and Glasgow Coma Scale (GCS) score at admission would be the most important significant predictors of physical and mental health outcomes.

## 2. Materials and Methods

### 2.1. Participants

This prospective longitudinal cohort study consisted of individuals with moderate to severe TBI who were admitted to the Trauma Referral Centre in Oslo, Norway, in 2005–2007. The participants were assessed in the acute phase (baseline) and followed up at 1, 2, 5, and 10 years after injury. The inclusion criteria were (a) age 16–55 years; (b) admission with an International Classification of Diseases 10th Revision (ICD-10) diagnosis S06.0–S06.9 within 24 h of injury; (c) moderate to severe TBI, classified by an acute GCS score of 3–12 [32] at admission or before intubation; and (d) residence in eastern Norway. The exclusion criteria were (a) previous neurological disorders/injuries; (b) associated spinal cord injuries; (c) previously diagnosed severe psychiatric or substance-abuse disorders; and (d) unknown address or incarceration.

In total, 133 individuals with TBI met the inclusion criteria. Thirty-two died during the study period and four dropped out before the 1-year follow-up. Here, we analyzed the trajectories of all 97 participants with a full data set at the 1-year follow-up. Due to further dropouts and a few missing data points, we had SF-36 data on 91–92 participants (one participant had a missing score on the Bodily Pain subscale) at the 2-year follow-up, 90 participants at the 5-year follow-up, and 73 participants at the 10-year follow-up. The attrition rate from the 1- to 10-year follow-up was 24.7%. The socio-demographics and injury severity characteristics are presented in Table 1.

### 2.2. Measures

The main outcome measure was the Medical Outcomes 36-Item Short Form Health Survey (SF-36) version 1 [7]. The SF-36 is a validated and reliable generic measure across different health conditions, including TBI [4,8,9]. The SF-36 consists of eight subscales: Physical Function (PF), Role Physical (RP, role limitations in daily activities due to physical health), Bodily Pain (BP), General Health (GH), Vitality (VT), Social Function (SF), Role Emotional (RE, role limitations in daily activities due to emotional health), and Mental Health (MH). Subscale raw scores were transformed into a scale score from 0 to 100 (worst to best). A mean difference of ≥5 in an SF-36 subscale score is regarded as clinically significant [33,34,35]. The subscales were weighted and summarized into the Physical Component Summary (PCS) score (PF, RP, BP, and GH) and the Mental Component Summary (MCS) score (VT, SF, RE, and MH). The PCS and MCS were scored using norm-based methods into standardized T-scores (mean value 50 ± 10). A license from Quality Metric Health Outcomes was used when scoring the 10-year data (license number QM036704). The PCS and MCS were used as the outcome measures of physical and mental health, respectively, and will be referred to accordingly throughout the article. The internal consistency and reliability of the PCS and MCS were measured with Cronbach’s alpha at each follow-up time point. The Cronbach’s α’s for the PCS and MCS subscales were 0.71–0.79 and 0.72–0.83, respectively, between the 1- and 10-year follow-ups, and were thus satisfactory (Cronbach’s α > 0.70).

The independent variables (predictors) were gender (male vs. female); age at time of injury (continuous, in years); relationship status at hospital admission (partnered (married/cohabitant) vs. single); education at admission (continuous in years or categorical, i.e., ≤12 years vs. >12 years); employment status at admission (employed vs. unemployed); occupation type at admission (blue collar (physical work) vs. white collar (non-physical work/student)); acute GCS score (continuous, range 3–12); cause of injury (traffic accident vs. other); duration of post-traumatic amnesia (PTA; continuous, in number of days); computed tomography (CT) head Marshall scores (grading injury severity from I (no visible intracranial pathology) to VI (non-evacuated mass lesions)) [36] on the “worst” CT scan within the first 24 h of injury; and Injury Severity Score (ISS, continuous, range 1–75 (best to worst)) [37].

### 2.3. Procedure

The pre-injury and injury-related characteristics were extracted from medical records. At the 1-, 2-, 5-, and 10-year follow-ups, most of the participant assessments were conducted by a physiatrist at the outpatient department. In some instances, the assessments were completed by an ambulatory team from the outpatient department, or by phone interview, if requested by the participants. 

### 2.4. Statistical Analysis

The changes in SF-36 subscales over time were described using descriptive analyses, and paired samples *t*-tests were run to examine the changes in subscales between the 1- and 10-year follow-ups. The predictive effect of the baseline demographic and injury characteristics on the PCS and MCS trajectories across 1, 2, 5, and 10 years after injury was assessed using hierarchical linear modelling (HLM). For each outcome, a conditional (null) model was run first to determine whether there was sufficiently large clustering of the PCS or MCS score variance within participants to proceed with HLM. Unconditional growth linear (straight line), quadratic (U-shaped), and cubic (S-shaped) models were then run with no predictors to determine the most accurate model for linear or polynomial curvature of the PCS and MCS scores over time.

Once the most accurate curvature model was identified for each outcome, the predictors were entered simultaneously as fixed effects into an HLM after being centered or given a reference point of 0, along with time. The first full model for each outcome used HLM to determine whether the PCS and MCS trajectories across the four time points could be predicted by the demographic and injury characteristics of time (coded as 0 (1 year), 1 (2 years), 4 (5 years), or 9 (10 years) to reflect the actual spacing between time points); gender (1 = female, 0 = male); age; relationship status (1 = partnered, 0 = single); education; employment at admission (1 = employed, 0 = unemployed); occupational status (1 = white collar, 0 = blue collar); GCS score; cause of injury (1 = motor vehicle, 0 = not motor vehicle); length of PTA (days); CT severity score; and ISS. A “trajectory” is both an intercept (overall height of a line) and slope (rate of increase or decrease of a line). Significant main effects in this model would reflect y-intercept differences as a function of the predictor, or in other words, differences in the heights of the overall regression lines over time. A final HLM for each outcome included the previously significant predictors from the first full model, time, as well as the interaction terms between the previously significant predictors and time. Significant interaction effects in these models would reflect slope differences as a function of the predictor, or in other words, differences in the slopes of the regression lines over time.

## 3. Results

### 3.1. SF-36 Subscale and Component Summary Score Changes over Time

**Figure 1:**
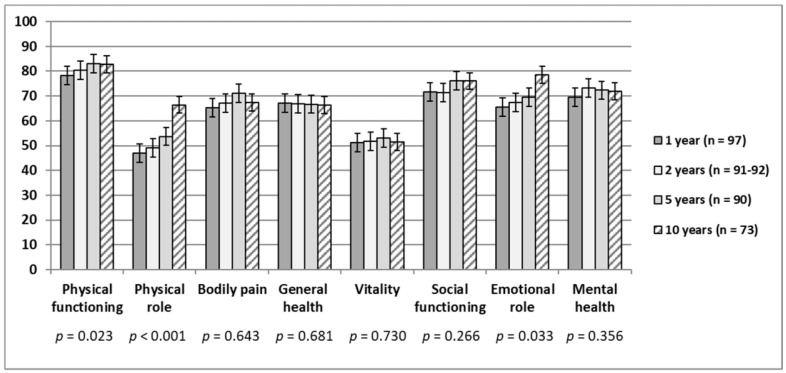
Medical Outcomes 36-Item Short Form Health Survey (SF-36) mean subscale score changes for the study population across 10 years after moderate to severe TBI. The error bars display the standard error of the mean subscale score at each time point. The *p*-values from paired samples *t*-tests investigating the change in mean scores from 1- to 10-year follow-up are shown below each subscale.

The eight SF-36 subscales in general showed either a stable trend or improvement up to 10 years after injury (Figure 1). Clinically relevant positive changes (mean difference ≥ 5) only occurred in two subscales between the 1- and 10-year follow-ups: RP and RE (19.5 and 12.9, respectively), with the largest change taking place between the 5-and 10-year follow-ups. PF and BP showed an improvement up to 5 years after injury, followed by a small decrease between 5 and 10 years. GH and VT remained almost unchanged throughout the period, with VT showing the lowest mean scores of all subscales (mean scores 51.3–53.0). SF remained relatively stable at the 1- to 2-year follow-ups, increasing from the 2- to 5-year follow-ups before plateauing up to the 10-year follow-up. MH showed a small increase from the 1- to 2-year follow-ups, before remaining relatively stable up to 10 years. The paired samples *t*-tests showed statistically significant changes in the subdomains of PF (*p* = 0.023), RP (*p* < 0.001), and RE (*p* = 0.033) from the 1- to 10-year follow-ups. The study population showed lower subscale scores across all eight subscales at 10 years as compared to the adjusted scores of the general population of Norway [38], where all subscale scores except PF showed a difference ≥5.

The PCS and MCS both showed a steady improvement over time. The PCS mean score increased from 43.0 at 1 year to 43.2, 44.8, and 48.6 at the 2-, 5-, and 10-year follow-ups, respectively. Similarly, the MCS mean score improved from 44.7 at 1 year to 45.5, 45.7, and 48.0 at the 2-, 5-, and 10-year follow-ups, respectively. The paired samples *t*-tests showed a significant change in both PCS and MCS mean scores between the 1- and 10-year follow-up (*p* < 0.001 and *p* = 0.006, respectively).

### 3.2. Physical Health (PCS) Trajectories

#### 3.2.1. Unconditional and Unconditional Growth Models

The unconditional model yielded a statistically significant estimated participant variance of 59.44 (Wald Z = 5.46, *p* < 0.001) and a statistically significant estimated residual variance of 47.76 (Wald Z = 11.01, *p* < 0.001). The intraclass correlation coefficient was calculated to be 0.55, indicating that approximately 55% of the total variance of the PCS was associated with the participant grouping and that the assumption of independence was violated. This suggests that there was sufficiently large clustering of PCS variance within the participants to proceed with HLM. The unconditional model was then run separately with the successive additions of time, quadratic time, and cubic time to determine the shape of the best-fitting curve of the PCS over time (Table 2), suggesting that a linear, or straight, trajectory best fit the PCS over time.

#### 3.2.2. Full Models

**Figure 2:**
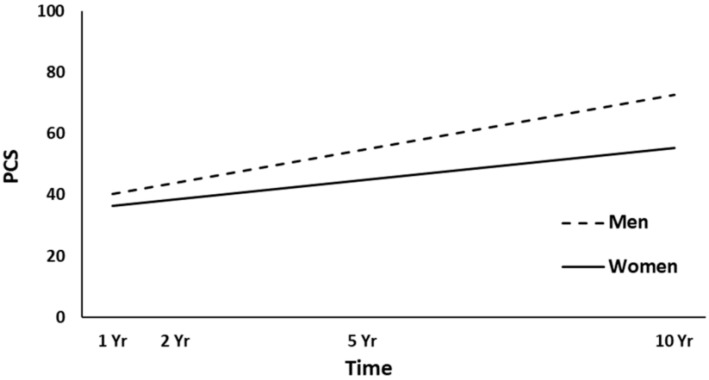
Main effect of gender on the physical health (PCS) trajectories. yr, year.

**Figure 3:**
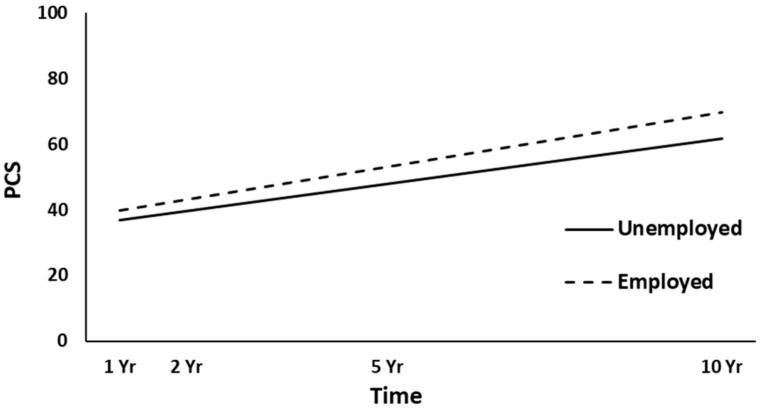
Main effect of employment at admission on the physical health (PCS) trajectories. yr, year.

**Figure 4:**
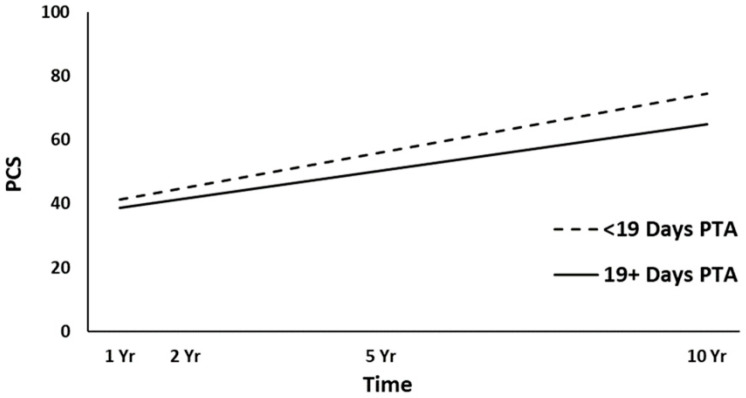
Main effect of post-traumatic amnesia (PTA) on the physical health (PCS) trajectories. yr, year.

The full HLM examined whether the height (intercept) of linear PCS trajectories over time could be predicted by the demographic and injury characteristics at baseline. Table 3 shows all the statistically significant and non-significant fixed effects from the full HLM and their b-weights, *p*-values, and 95% confidence intervals. In the main effect model of the PCS trajectories, time, gender, employment at admission, and PTA yielded statistically significant effects. Across the four time points, the PCS scores were significantly increased (*p* < 0.001). Women had lower overall PCS trajectories than men (*p* < 0.001, Figure 2). Participants employed pre-injury (*p* = 0.016, Figure 3) and those with a shorter duration of PTA (*p* = 0.031, Figure 4) had higher PCS trajectories over time. Occupational status approached the significance level (*p* = 0.052), with those in white-collar positions tending to show higher PCS trajectories than those in blue-collar positions. No other predictors were statistically significant (all, *p* ≥ 0.052). A final HLM revealed that the interaction terms between time and the previously significant predictors were not significant, suggesting that the PCS trajectories did not change differentially over time (i.e., a different slope) as a function of gender, employment at admission, or PTA.

### 3.3. Mental Health (MCS) Trajectories

#### 3.3.1. Unconditional and Unconditional Growth Models

The unconditional model yielded a statistically significant estimated participant variance of 67.83 (Wald Z = 5.29, *p* < 0.001) and a statistically significant estimated residual variance of 66.86 (Wald Z = 11.06, *p* < 0.001). The intraclass correlation coefficient was calculated to be 0.50, indicating that approximately 50% of the total variance of the MCS was associated with the participant grouping, and again that the assumption of independence was violated. The unconditional model was then run separately with the successive additions of time, quadratic time, and cubic time to determine the shape of the best-fitting curve of the MCS over time (Table 2), suggesting that, as with the PCS, a linear trajectory best fit the MCS over time.

#### 3.3.2. Full Models

**Figure 5:**
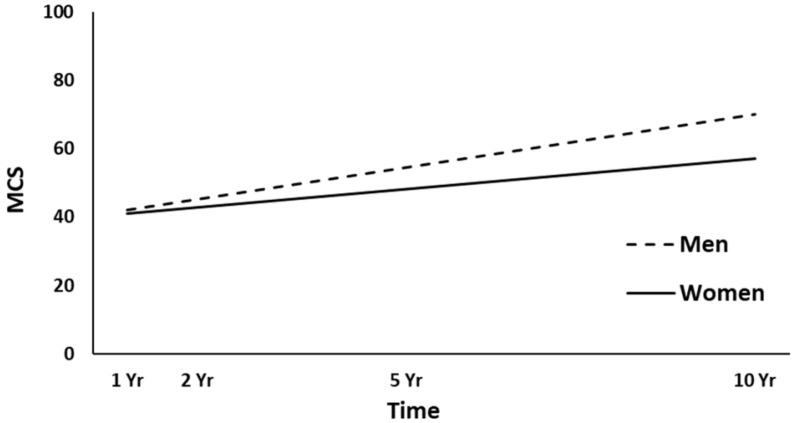
Main effect of gender on the mental health (MCS) trajectories. yr, year.

**Figure 6:**
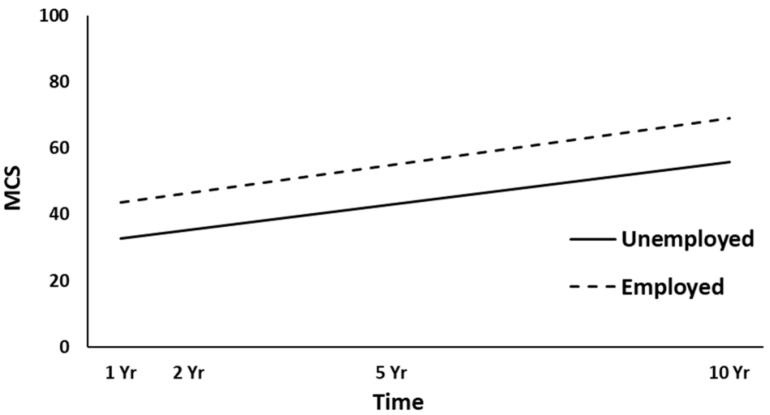
Main effect of employment at admission on the mental health (MCS) trajectories. yr, year.

The full HLM examined whether the height (intercept) of the MCS trajectories over time could be predicted by demographic and injury characteristics at baseline. Table 4 shows all the statistically significant and non-significant fixed effects from the full HLM and their b-weights, *p*-values, and 95% confidence intervals. In the main effect model of the MCS trajectories, time, gender, and employment at admission yielded statistically significant effects. Across the four time points, MCS scores were significantly increased (*p* = 0.006). Women had lower overall MCS trajectories than men (*p* = 0.001, Figure 5), and participants employed pre-injury (*p* < 0.001, Figure 6) had higher MCS trajectories over time. ISS approached significance (*p* = 0.051), whereas no other predictors were statistically significant (all, *p* > 0.051). A final HLM revealed that the interaction terms between time and the previously significant predictors were not significant, suggesting that the MCS trajectories did not change differentially over time (i.e., a different slope) as a function of gender or employment at admission.

## 4. Discussion

In the present study, we investigated the longitudinal trajectories of HRQL over the first decade after moderate to severe TBI. The rehabilitation and recovery process after sustaining moderate to severe TBI has been described as continuous, complex, and dynamic [39]. In addition, a person’s life situation will change over time with regard to a number of factors, for example, work, friends and social support, marital status, family life and dependency of children, and impact of other health conditions [1,40]. All these factors can influence a person’s physical, mental, emotional, and social functioning; thus, the HRQL could change over time. The HLM method, complementary to previous HRQL research using a cohort or cross-sectional design with one follow-up time point [10,11,13,14,15,41,42], allowed the evaluation of more complex HRQL trajectories during a 10-year period after TBI as well as the relationships between injury severity and demographic predictors in the acute period.

### 4.1. Changes in SF-36 Subscales and Component Summary Scores

We hypothesized that the SF-36 subscale scores and component summary scores would increase over time, and our results partly support this hypothesis. The mean scores of the eight SF-36 subscales over 1, 2, 5, and 10 years after injury displayed either a stable trend or an improvement. The PF, RP, and RE subscales showed significant increases from 1 to 10 years, although only RP and RE showed clinically relevant positive changes with a scale difference of ≥5. These subscales relate to role limitations due to problems with physical and emotional functioning, and the positive change between the 5- and 10-year follow-ups could be due to an actual improvement in functioning, or due to a response shift within the participants, or a combination of both. Response shift refers to the change of internal standards, values, expectations, and goals due to an external factor or event, such as a change in health status (e.g., sustaining a TBI). The patient creates a new narrative of their life, including their self-view and values, and may then set new goals based on adjusted and (hopefully) realistic expectations. In other words, the person may change the foundation on which they assess quality of life over time. As some of the questions pertaining to the SF-36 RP and RE subscales are more open to interpretation and evaluation based on one’s expectations (e.g., “accomplished less than you would like”) than other subscales such as PF with more concrete questions (e.g., “walking one block”), these two subscales might be more susceptible to response shift, or a different way of thinking about impairments, than others.

A systematic review on HRQL after TBI by Polinder et al. [4] determined that people with TBI still showed large deficits from full recovery compared to population norms in the long-term. Meta-analysis revealed that the RP and VT subscales showed the lowest scores in general; however, the RP, RE, and SF subscales had the lowest scores for individuals with TBI compared to the population norm scores [4]. In a longitudinal study, Grauwmeijer et al. [17,18] examined HRQL up to 10 years after moderate to severe TBI, and found that the subscale scores reached the norm of the general Dutch population by year 3, but they did not find any significant further changes in subscale scores between the 3- and 10-year follow-ups. The subscale scores reported by Andelic et al. [10] in another Norwegian 10-year cohort are very similar to those in the present study, where both studies in general show lower HRQL than reported in the general population [38].

As the subscale scores showed a positive change from the 1- to 10-year follow-ups, the PCS and MCS accordingly both showed steady improvement over time in a linear fashion. The PCS mean score showed a significant change from 43.0 at 1 year to 48.6 at the 10-year follow-up, while the MCS mean score similarly showed a significant change from 44.7 at 1 year to 48.0 at the 10-year follow-up. It is worth mentioning that the physical and mental health summary scores across the follow-ups were >40 (<1 standard deviation below the general population norms), indicating good health [30]. For comparison, Grauwmeijer et al. reported a change in PCS from 42 (1-year follow-up) to 45 (10-year follow-up), and in MCS from 49 (1-year follow-up) to 51 (10-year follow-up) in a Dutch TBI population [17,18]. Regarding cross-sectional studies, Jacobsson et al. [15] reported a PCS of 42 and MCS of 48 in 67 individuals on average 10 years after TBI in Sweden, whereas Cantor et al. [41] reported a PCS of 41.2 and MCS of 42.7 in 223 individuals on average 15 years after TBI in the US. In the last two studies, however, the large range in follow-up time might overshadow possible time-specific changes long-term.

### 4.2. Predictors of Physical and Mental Health Trajectories

Regarding the possible predictors of the physical and mental health trajectories up to 10 years after injury, we hypothesized based on previous research that employment pre-injury, gender, and GCS would be significant predictors of HRQL. The analyses of the physical health trajectories showed that gender, employment pre-injury, and length of PTA were significant predictors, whereas occupational status approached the significance level. The analyses of the mental health trajectories showed that gender and employment pre-injury were the only significant predictors, whereas ISS approached the significance level. To sum up, our hypothesis was correct with the exception that length of PTA was a significant predictor instead of GCS.

Women showed lower overall trajectories for both physical and mental health, in line with several other studies reporting lower HRQL for women after TBI [10,16,25,27,28], and in contrast to other studies [15,21]. The results may reflect the trend of the Norwegian general population, where women overall report lower HRQL than men [38]. In the present study, the lack of association between age and HRQL could have been due to the limited age range (16–55 years). A younger age is often associated with higher HRQL [13,20,28]; however, some have reported the opposite [25] or no association [10,16]. Here, participants who were employed pre-injury showed higher physical and mental health trajectories over time, in line with an extensive literature showing a strong association between work and HRQL [10,15,16,21,23]. A previous study on the same cohort examining physical functioning domains in the SF-36 found that white-collar occupational status was associated with higher PF and GH scores at a 5-year follow-up after injury [31]. Those with shorter PTA (indicating less severe injury) showed higher physical health trajectories over time. Another Norwegian study did not find any association between PTA and HRQL at 22 years after hospitalization for TBI [13]. In the present study, neither the GCS nor CT severity score were significant predictors of HRQL. Other studies have found an association between the GCS score and short-term HRQL [20,27,28], whereas several studies found no association between injury severity and long-term HRQL after injury [10,11,15,16,18]. Although the ISS approached significance for the mental health trajectories, it did not predict the physical health trajectories in the present sample. In contrast, a previous study on the same cohort showed that the ISS was a significant predictor of better physical health (PCS) in the first years after TBI [20]. Hence, the results from the present study may suggest that the influence of both TBI severity and overall trauma severity on physical health becomes smaller in the long term.

A German study investigating HRQL in isolated TBI vs. TBI with polytrauma found no difference in the SF-36 subscale scores between the groups except for PF at 1 year post-injury [43]. However, an impact of ISS on PCS rather than MCS would seem more probable, as more severe polytrauma can lead to worse physical function and greater limitations due to the accompanying injuries, especially in the acute phase but also over time, as well as possible development of secondary chronic pain conditions. The HRQL 10-year outcomes reported in a primary polytrauma population support these speculations, where physical health was reduced compared with that of the adjusted general population at 10 years after injury, whereas mental health did not differ from that of the general population [44]. However, based on the present study results, more severe polytrauma alongside TBI does not have a negative impact on physical health trajectories long-term after injury.

### 4.3. Limitations, Strengths, and Future Directions

The present study has strengths and limitations that the reader should take into consideration when evaluating the results. The participants were recruited through the Trauma Referral Centre for the Southeast region (the largest region) of Norway, and therefore represent a mixed population with different levels of received inpatient rehabilitation that increases the generalizability of the findings. The use of HLM with a longitudinal design with four consecutive follow-ups provides stronger statistical power for analyses, despite a small study sample, through increasing the number of observations from 97 (i.e., one time point) to 388 (i.e., four time points). The inclusion criteria eliminated individuals with mild TBI or age >55 or <16 years in the study population, which consequently hinders generalizability of the findings to those with mild TBI and those with a higher/lower age at the time of injury.

Recovery, acceptance, and adjustment to life after TBI may take many years. Future long-term studies should be conducted to ensure that the findings are generalizable to other countries, and to incorporate the relevant variables beyond socio-demographic and injury-related characteristics. For example, future studies should take into account the impact of physical and cognitive functioning, emotional functioning (i.e., depression), employment, social participation, social support, and subjective factors such as personality traits, resilience, self-awareness, and response shift. In addition, the use of the SF-36 in combination with a TBI-specific instrument, i.e., Quality of Life after Brain Injury (QOLIBRI), as recommended by Polinder et al. [4], will cover important information specifically relevant to individuals with TBI and result in further-improved knowledge of the long-term recovery and consequences after TBI [5,45].

## 5. Conclusions

Most SF-36 subscale scores showed a stable or increasing trend over the first 10 years after injury, with RP and RE being the only subscales with clinically relevant positive changes. The physical (PCS) and mental (MCS) health trajectories increased in a linear fashion over time. Female gender, pre-injury unemployment, and more severe injuries (longer PTA) predicted less favorable HRQL trajectories. At-risk individuals displaying these characteristics may be targeted to receive regular follow-up to improve their quality of life outcomes, and the findings highlight the importance of long-term care.

## Figures and Tables

**Table 1:** Socio-demographics at the time of injury and the injury characteristics.

Variable	*n* (%)	Total *n*
Age at injury		97
Mean (SD)	30.3 (10.8)	
Gender		97
Male	76 (78.4)	
Female	21 (21.6)	
Relationship status		97
Partnered	28 (28.9)	
Single	69 (71.1)	
Education level		96
≤12 years	54 (56.3)	
>12 years	42 (43.8)	
Employment status		97
Employed	80 (82.5)	
Unemployed	17 (17.5)	
Occupation type		97
Blue collar	46 (47.4)	
White collar	51 (52.6)	
Injury cause		97
Traffic accident	58 (59.8)	
Fall	23 (23.7)	
Violence/other	16 (16.5)	
Glasgow Coma Scale score		97
Mean (SD)	7.2 (3.2)	
Moderate (9–12)	32 (33.0)	
Severe (3–8)	65 (67.0)	
Post-traumatic amnesia duration		91
Days, Mean (SD)	26.0 (30.0)	
CT Head Marshall Score		97
Score I	15 (15.5)	
Score II	34 (35.1)	
Score III	38 (39.2)	
Score IV	1 (1.0)	
Score V	9 (9.3)	
Score VI	0 (0.0)	
Injury Severity Score		97
<16	15 (15.5)	
≥16	82 (84.5)	

**Table 2:** Model fit for the Physical Component Summary (PCS) and Mental Component Summary (MCS) trajectories over time.

Model	−2 Log Likelihood
PCS	
Unconditional Growth Model	2380.28
Quadratic	2378.31
Cubic	2378.08
MCS	
Unconditional Growth Model	2513.52
Quadratic	2513.07
Cubic	2513.01

Note: The critical χ2 value for significant difference at α = 0.05 is a > 3.841 drop from the previous model.

**Table 3:** Socio-demographic and injury predictors of the physical health (PCS) trajectories across 1, 2, 5, and 10 years.

Predictor	b-Weight	SE	*p*-Value	95% CI	
				**Lower Bound**	**Upper Bound**
Intercept	39.03	2.26	0.000 **	34.53	43.52
Time	0.65	0.11	0.000 **	0.44	0.86
Gender (1 = woman, 0 = man)	−7.22	1.87	0.000 **	−1.94	−3.50
Age	−0.12	0.09	0.194	−0.29	0.06
Relationship Status (1 = partnered, 0 = single)	−1.55	2.03	0.448	−5.59	2.49
Education	0.67	1.03	0.518	−1.38	2.71
Employment (1 = employed, 0 = unemployed)	4.98	2.04	0.016 *	0.94	9.02
Occupational Status (1 = white collar, 0 = blue collar)	3.48	1.77	0.052	−0.03	6.99
Glasgow Coma Scale Score	0.03	0.28	0.926	−0.54	0.59
Cause of Injury (1 = motor vehicle, 0 = not motor vehicle)	−1.10	1.76	0.532	−4.60	2.39
Post-Traumatic Amnesia	−0.07	0.03	0.031 *	−0.13	−0.01
CT Severity Score	1.20	0.77	0.121	−0.32	2.72
Injury Severity Score	0.07	0.06	0.293	−0.06	0.19

Note. * = *p* < 0.05; ** = *p* < 0.001. SE = standard error. CI = confidence interval.

**Table 4:** Socio-demographic and injury predictors of the mental health (MCS) trajectories across 1, 2, 5, and 10 years.

Predictor	b-Weight	SE	*p*-Value	95% CI	
				**Lower Bound**	**Upper Bound**
Intercept	33.35	2.69	0.000 **	28.01	38.68
Time	0.37	0.14	0.006 *	0.11	0.64
Gender (1 = woman, 0 = man)	−7.60	2.21	0.001 *	−12.00	−3.20
Age	−0.13	0.11	0.230	−0.34	0.08
Relationship Status (1 = partnered, 0 = single)	3.42	2.41	0.159	−1.36	8.21
Education	−1.69	1.22	0.168	−4.12	0.73
Employment (1 = employed, 0 = unemployed)	11.10	2.41	0.000 **	6.30	15.90
Occupational Status (1 = white collar, 0 = blue collar)	3.36	2.09	0.112	−0.80	7.51
Glasgow Coma Scale Score	0.08	0.34	0.819	−0.59	0.74
Cause of Injury (1 = motor vehicle, 0 = not motor vehicle)	−0.83	2.08	0.692	−4.97	3.31
Post-Traumatic Amnesia	0.02	0.04	0.652	−0.05	0.09
CT Severity Score	−0.33	0.91	0.721	−2.13	1.48
Injury Severity Score	0.15	0.08	0.051	0.00	0.30

Note: * = *p* < 0.01; ** = *p* < 0.001. SE = standard error. CI = confidence interval.

## Data Availability

The data presented in this study may be available on request from the corresponding author, if considered appropriate and approved by the Regional Committees for Medical and Health Research Ethics (REC). The data are not publicly available due to lack of anonymity in accordance with the Norwegian law on research ethics and medical research.
